# Towards elimination of mother-to-child transmission of HIV: performance of different models of care for initiating lifelong antiretroviral therapy for pregnant women in Malawi (Option B+)

**DOI:** 10.7448/IAS.17.1.18994

**Published:** 2014-07-28

**Authors:** Monique van Lettow, Richard Bedell, Isabell Mayuni, Gabriel Mateyu, Megan Landes, Adrienne K Chan, Vanessa van Schoor, Teferi Beyene, Anthony D Harries, Stephen Chu, Andrew Mganga, Joep J van Oosterhout

**Affiliations:** 1 Dignitas International, Zomba, Malawi; 2 Dalla Lana School of Public Health, University of Toronto, Toronto, Canada; 3 Department of Family and Community Medicine, University of Toronto, Toronto, Canada; 4 Division of Infectious Diseases, Sunnybrook Health Sciences Centre, Toronto, Canada; 5 International Union Against Tuberculosis and Lung Disease, Paris, France; 6 Department of Clinical Research, London School of Hygiene and Tropical Medicine, London, UK; 7 Zonal Health Office, Malawi Ministry of Health, Zomba, Malawi; 8 Department of HIV and AIDS, Malawi Ministry of Health, Lilongwe, Malawi

**Keywords:** PMTCT, Option B+, Malawi, service delivery model, model of care, retention

## Abstract

**Introduction:**

Malawi introduced a new strategy to improve the effectiveness of prevention of mother-to-child HIV transmission (PMTCT), the Option B+ strategy. We aimed to (i) describe how Option B+ is provided in health facilities in the South East Zone in Malawi, identifying the diverse approaches to service organization (the “model of care”) and (ii) explore associations between the “model of care” and health facility–level uptake and retention rates for pregnant women identified as HIV-positive at antenatal (ANC) clinics.

**Methods:**

A health facility survey was conducted in all facilities providing PMTCT/antiretroviral therapy (ART) services in six of Malawi's 28 districts to describe and compare Option B+ service delivery models. Associations of identified models with program performance were explored using facility cohort reports.

**Results:**

Among 141 health facilities, four “models of care” were identified: A) facilities where newly identified HIV-positive women are initiated and followed on ART at the ANC clinic until delivery; B) facilities where newly identified HIV-positive women receive only the first dose of ART at the ANC clinic, and are referred to the ART clinic for follow-up; C) facilities where newly identified HIV-positive women are referred from ANC to the ART clinic for initiation and follow-up of ART; and D) facilities serving as ART referral sites (not providing ANC). The proportion of women tested for HIV during ANC was highest in facilities applying Model A and lowest in facilities applying Model B. The highest retention rates were reported in Model C and D facilities and lowest in Model B facilities. In multivariable analyses, health facility factors independently associated with uptake of HIV testing and counselling (HTC) in ANC were number of women per HTC counsellor, HIV test kit availability, and the “model of care” applied; factors independently associated with ART retention were district location, patient volume and the “model of care” applied.

**Conclusions:**

A large variety exists in the way health facilities have integrated PMTCT Option B+ care into routine service delivery. This study showed that the “model of care” chosen is associated with uptake of HIV testing in ANC and retention in care on ART. Further patient-level research is needed to guide policy recommendations.

## Introduction

In 2011, Malawi introduced a new strategy to improve the effectiveness of its prevention of mother-to-child HIV transmission (PMTCT) program by providing a standardized combination antiretroviral therapy (ART) regimen to all HIV-positive pregnant and breastfeeding women, irrespective of their CD4 count or clinical stage of HIV infection, and that ART be continued lifelong. This was an extension of an existing WHO recommendation for PMTCT (Option B) and Malawi's strategy was therefore dubbed Option B+. This public health approach for PMTCT has since been adopted by several other countries in the region and was included in the updated WHO guidelines in 2013 [1]. The Option B+ strategy has also received criticism citing lack of a firm evidence base, including unstudied effects of long-term ART in otherwise healthy women and infants, and potential for increased drug resistance with poor adherence [2,3].

Upon initiation of the new strategy, practical issues about how to organize HIV care and ART provision for women in antenatal care (ANC) arose across the spectrum of often poorly staffed health facilities in Malawi. Despite these challenges, initial reports demonstrated that the Option B+ strategy has resulted in large increases in the number of pregnant women accessing PMTCT [4]. However, concern has been raised about the level of attrition of women from care and it has been noted that large variations exist in rates of retention between health facilities [5].

Clearly, more information is needed about the factors that determine retention and attrition in pregnant women who start ART under the Option B+ strategy.

From July 2011 onwards, health facilities in Malawi were tasked with integrating Option B+ into the routine service delivery and as this was uncharted territory, the Malawi Ministry of Health did not provide specific guidelines for how this should be done. Different approaches had to be considered which would account for the location of ART initiation (at the ANC or ART clinic), the timing of ART initiation after a positive HIV test result, the timing and type of adherence counselling for ART initiation, the location of follow-up after delivery and/or breastfeeding, and the type of support women receive. There was no evidence about how these different approaches would affect acceptability, uptake, adherence and retention in care over the course of pregnancy, during lactation, and post-lactation.

The aim of this study was to describe how the PMTCT/Option B+ services are being delivered to pregnant women in health facilities in the South-East Zone in Malawi, identifying the diversity in approaches to service organization (the “model of care”). We also aimed to explore associations between the “model of care” and national program indicators at health facility level, in particular uptake of HIV testing in ANC, and uptake of and retention in care on ART.

## Methods

### Malawi National ART/PMTCT program

The scale-up of free ART started in 2004 using standard ART regimens and relying mainly on clinical monitoring for toxicity and treatment failure. The program has a monitoring and evaluation framework of high quality [6]. PMTCT was started in 2002, and evolved from the use of single dose nevirapine (sdNVP) for mother and infant, to combination prophylaxis with twice daily zidovudine (AZT) for the mother during pregnancy with the addition of sdNVP during labour, a one week AZT-lamivudine (3TC) tail, and one–four week prophylaxis with AZT-syrup for the infant) [7] implemented since 2007, but only in the larger health facilities in the country. In 2011, the Option B+ strategy was initiated using a single-tablet fixed dose combination of tenofovir, lamivudine and efavirenz [8]. The sdNVP regimen and the AZT combination prophylaxis were phased out by April 2012. By the end of June 2013, there were 588 sites enrolling women under PMTCT Option B+ [9].

### South East Zone of Malawi

Malawi is divided into 28 districts and organized into five health zones. The South East Zone includes six districts and has approximately 3.5 million inhabitants (23% of the total population of 14.8 million in 2012). There are around 154,000 pregnancies in the South East Zone per annum and 22,500 (14.6%) of these are among HIV-positive women [10].

By June 2013, the South East Zone counted 153 facilities with integrated HIV care services, of which 94% provided PMTCT Option B+ [9]. The Canadian non-governmental organization Dignitas International has been operating in partnership with the Malawi Ministry of Health since 2004, initially in Zomba District, and expanding throughout the South East Zone from 2010 to support the delivery of HIV-related services through training, mentorship and supervision.

### Facility-level data: study methodology, data collection, definitions and statistical analysis

A survey was conducted in all health facilities providing PMTCT/ART services in the South East Zone to describe PMTCT/Option B+ service delivery models. Associations were explored between these models and the uptake of HIV testing and counselling (HTC) and ART among pregnant women, as well as the six- and 12-month treatment outcomes among women registered as having started ART under Option B+.

### Facility survey

Data on the organization of Option B+ service delivery were collected between February and June 2013, using a structured questionnaire administered to the health facility officer in-charge. It included questions regarding staff involved in PMTCT/ART provision, availability of ANC/ART services (offered all weekdays or not), location where newly HIV-positive women are initiated on ART (at ANC or ART clinic), timing and type of adherence counselling for ART initiation (same day as ART initiation, during next visit after ART initiation or both, and in group or individual sessions) and the timing of transfer to ART or mother–infant-pair (MIP) clinic. Facilities that indicated that their organizational arrangement had changed within the past six months were excluded from the analysis.

### Facility-level cohort data

Routinely collected health facility cohort reports that are validated quarterly were used to ascertain uptake of HTC and ART initiation for pregnant women with newly identified HIV infections in ANC between July 2012 and June 2013. These included information about HIV test kit stock outs. Routinely collected cohort survival outcome data were used to evaluate outcomes for women registered as having started ART under Option B+. Cohort reports of women who started between July and December 2012 were used for six-month outcomes; and reports of those who started between January and June 2012 were used for 12-month outcomes. To explore changes over calendar time, six-month outcomes were also ascertained for women who started between January and June 2012.

Retention was defined as the number of women retained on ART (by six or 12 months) divided by the number of women in the cohort minus the number of women who transferred out during the observation period. As per the national guideline, women on ART were counted as “defaulted” if they were expected to have run out of ARVs for two or more months (based on the number of tablets given at the last visit) and were not known to have transferred out, stopped or died [11].

### Statistical analysis

Data analyses were conducted using IBM SPSS Statistics 20 (IBM, Armonk, NY, USA). Health facility characteristics were described with proportions and 95% confidence intervals [CI] or medians with interquartile ranges [IQR]. Four PMTCT Option B+ service delivery models were identified through the descriptive analysis of the health facility survey and were used as categories for further analysis. Comparisons between groups were made using the Chi-square test, nonparametric independent sample median tests and analysis of variance.

To explore associations between service organization and uptake of HTC during ANC at facility level, binary logistic regression models were fitted with the proportion of women with HIV status ascertained >85% (above the national target for 2012–2013[12]) as the outcome variable.

To explore associations between service organization and retention rates at health facility level, multinomial logistic regression models were fitted with “six-month retention rate” at health facility level as the outcome variable. In the absence of a widely accepted definition of a good/acceptable/bad six-month retention rate, we used the IQR as the reference category in these analyses, with low retention defined as below the IQR and high retention defined as above the IQR. Continuous predictor variables were similarly categorized, except that the category below the IQR was used as the reference category, and the other categories were entered in the model in order of increased retention.

Crude odds ratios (OR) and adjusted odds ratios (aORs) with 95% CI's were calculated for each model and were controlled for all the variables in the model, namely district, facility type, PMTCT/ART services availability, number of women registered or in the Option B+ cohort, client provider ratios and model of Option B+ provision as defined in results section. In addition, quarterly HIV test kit stock outs were included in the model exploring associations with uptake of HTC, and timing of adherence counselling and availability of ART/MIP clinics for follow-up were included in the model exploring associations with retention rates. A significance level of 0.05 was set for all statistical testing.

### Ethical approval

Ethical approval was granted by the National Health Sciences Research Committee, Malawi (#1084) and the Ethics Advisory Group of the International Union Against Tuberculosis and Lung Disease, Paris, France (#71/12).

## Results

### Study sites

Of 153 health facilities in the South East Zone providing PMTCT/ART services at the time of the survey, 141 were included in the study. Not included were Zomba Central Hospital, one of the four central referral hospitals in the country, 10 facilities that had only recently started providing PMTCT Option B+ services, and one health centre that was overlooked by the study team.

Table 1 gives an overview of the health facility characteristics.

**Table 1:** Health facilities, number of staff involved in PMTCT/ART provision, and number of women newly registered for ANC and ART initiation during ante-natal care

Expressed in median (IQR) or *Totals*	District hospital (*n*=4)	Community hospitals (*n*=8)	Health centres (*n*=120)	Private clinics (*n*=9)	Total (*n=*141)
Medical assistants	5 (1–25)	2 (2–4)	1 (1–2)	0 (0–0)	198
Nurses/midwives	40 (23–72)	19 (14–28)	2 (1–3)	3 (2–3)	712
Clinical officers	5 (2–9)	3 (1–5)	0 (0–0)	1 (1–2)	82
Medical doctors	2 (2–3)	0 (0–1)	0 (0–0)	0 (0–1)	20
HTC counsellors	14 (6–24)	8 (4–10)	4 (3–5)	2 (2–3)	614
ART initiation counsellors	5 (4–17)	3 (1–7)	1 (1–2)	0 (0–0)	262
Expert patients	2 (1–3)	2 (0–2)	1 (0–2)	0 (0–0)	188
ART clerks	4 (2–8)	2 (1–2)	2 (1–2)	1 (0–1)	280
Total number of staff	90 (60–139)	39 (31–46)	12 (9–15)	9 (8–11)	2356
Average number of women newly registered for ANC per quarter (based on quarterly data Jul 2012–Jun 2013)^a^	1025 (839–1318)	377 (282–538)	265 (146–373)	116 (7–225)	41,203
Average number of women newly tested positive at ANC per quarter (based on quarterly data Jul 2012–Jun 2013)^a^	79 (68–96)	21 (12–31)	11 (6–19)	6 (1–16)	2111
Number of women registered as having started ART (Option B+) between Jan–Jun 2012^b^	194 (90–298)	134 (59–151)	49 (27–89)	10 (3–20)	8864
Number of women registered as having started ART (Option B+) between Jul–Dec 2012^b^	184 (74–300)	105 (79–136)	50 (33–71)	14 (4–22)	9343

Note: ANC=antenatal care; ART=antiretroviral therapy.

a ANC not provided in one district hospital, three health centres and five private clinics;

b ART not provided in one private clinic.

### Identified models of care

**Figure 1:**
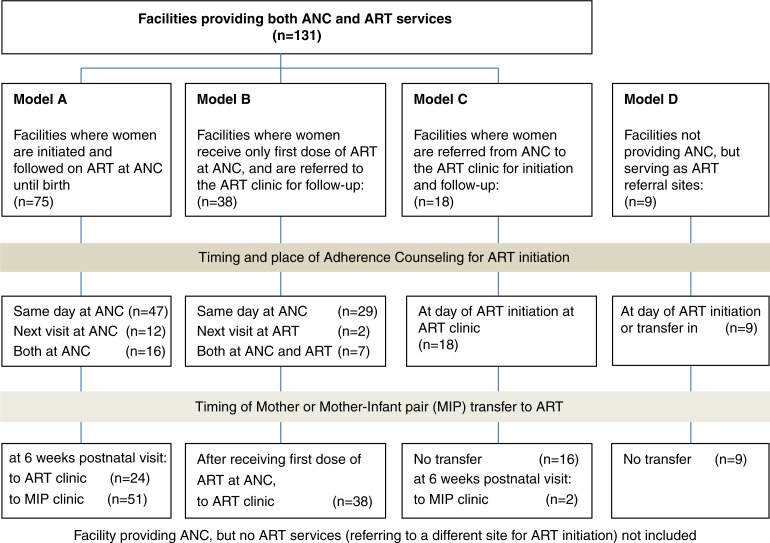
PMTCT service delivery models in health facilities in the South East Zone in Malawi.

The organization of Option B+ service delivery in the different facilities is presented in Figure 1.

#### Model A: Newly identified HIV-positive women are initiated and followed on ART at the ANC clinic until delivery

In 26 out of the 75 facilities applying Model A, ANC/ART services were offered on all weekdays and in 49 facilities on certain weekdays only. In the majority of facilities, adherence counselling was reported to be provided on the day of ART initiation only. Thirty-seven of the 75 facilities reported providing both individual and group counselling, 31 facilities provided individual counselling only. Seven of the 12 facilities that reported providing counselling during the next visit after ART initiation only, did so in group sessions only. All facilities transferred mother–infant pairs to the MIP or ART clinics at the six-week postnatal visit.

#### Model B: Newly identified HIV-positive women receive only the first dose of ART at the ANC clinic and are then referred to the ART clinic for follow-up

In 17 of the 38 facilities applying Model B, ANC/ART services were offered on all weekdays and in 21 facilities on certain weekdays only. In the majority of facilities, adherence counselling was reported to be provided on the day of ART initiation only, through individual counselling only (11 facilities), group counselling only (two facilities), or both (seven facilities).

#### Model C: Newly identified HIV-positive women are referred from the ANC clinic to the ART clinic in the same facility for initiation and follow-up of ART

Of the 18 facilities applying Model C, nine do so on a daily basis and nine less than daily, but in all facilities ART clinics are run on the same days as ANC clinics. Adherence counselling is expected to take place during ART initiation in the ART clinic.

#### Model D: Facilities that serve as ART referral sites and do not provide ANC

Pregnant and breastfeeding women may start and/or are followed on ART after referral from an ANC or Maternity Clinic in another site. These facilities reported providing adherence counselling at the time of ART initiation, one through both individual and group counselling, and eight facilities through individual counselling only.

### Performance of health facility by model of care

In Table 2, health facility performance is shown in relation to the organization of the Option B+ service delivery model.

**Table 2:** Health Facility Performance by PMTCT service delivery model

		Model A (*n*=73)^a^	Model B (*n*=36)^a^	Model C (*n*=18)	Model D (*n*=9)	*p*
Facility type	District hospitals	2	1	–	1	
	Community hospitals	5	–	3	–	
	Health centres	64	35	14	3	
	Private clinics	2	–	1	5	
ANC indicators (based on quarterly data July 2012–Jun 2013)	Average number of women newly registered for ANC per quarter, median (IQR)	252 (141–401)	316 (205–445)	224 (128–301)		0.07
	Average number of women known HIV-positive (previously tested positive), median (IQR)	5 (3–9)	4 (3–13)	4 (3–9)		0.79
	Average number of women newly tested positive, median (IQR)	11 (6–20)	12 (7–22)	10 (4–17)		0.42
	% of known HIV-positive women already on ART (started before ANC) (95% CI)	84 (79–89)	81 (75–87)	85 (76–94)		0.71
	% of HIV-positive women (not already on ART) started ART during ANC (95% CI)^b^	82 (76–87)	81 (74–89)	80 (68–91)		0.96
	% women not tested for HIV during ANC (95% CI)	18 (15–22)	32 (26–39)	30 (20–40)		0.001
Survival and retention						
6 month outcomes	Number of women in cohort, median (IQR)	52 (33–72)	55 (35–115)	45 (23–67)	26 (15–37)	0.13
Registered Jul–Dec 2012	% of women transferred out (95% CI)	8 (5–11)	5 (3–6)	6 (1–13)	10 (2–19)	0.49
Of those not transferred out	% retained on ART (95% CI)	80 (77–84)	79 (74–82)	89 (84–95)	92 (83–99)	0.008
	% not retained (95% CI)					
	Defaulted	18 (14–22)	20 (16–25)	10 (5–15)	7 (1–17)	0.02
	Stopped	0.4 (0–0.1)	0.01 (0–0.1)	0.01 (0–0.1)	–	0.62
	Died	1 (0–2)	0.01 (0–1)	1 (0–1)	0.02 (0–1)	0.58
Survival and retention						
12 month outcomes	Number of women in cohort, median (IQR)	52 (28–86)	51 (31–124)	28 (17–74)	21 (10–76)	0.87
Registered Jan–Jun 2012	% of women transferred out (95% CI)	8 (5–11)	7 (4–9)	4 (2–6)	10 (1–20)	0.42
Of those not transferred out	% retained on ART (95% CI)	80 (77–83)	77 (73–82)	87 (83–92)	94 (88–99)	0.002
	% not retained (95% CI)					
	Defaulted	18 (15–22)	22 (17–25)	12 (8–16)	6 (1–11)	0.006
	Stopped	1 (0–2)	0.01 (0–0.1)	1 (0–1)	–	0.55
	Died	1 (0–2)	0.01 (0–1)	1 (0–1)	0.3 (0.1–1)	0.70
Client to staff ratio	Number of women newly registered for ANC per Clinical Staff^c^ Member per month, median (IQR)	23 (10–33)	34 (24–43)	11 (5–26)	–	0.001
	Number of women newly registered for ANC per HTC Counsellor per month, median (IQR)	19 (10–23)	36 (19–47)	15 (10–27)	–	0.02
	Number of women in six month cohort per					
	Clinical Staff^c^ Member, median (IQR)	12 (6–20)	21 (11–29)	9 (3–13)	6 (3–10)	0.02

Note: Model A: Facilities where women are initiated and followed on ART at ANC until delivery; Model B: Facilities where women receive only first dose of ART at ANC, and are referred to the ART clinic for follow-up; Model C: Facilities where women are referred from ANC to the ART clinic for initiation and follow-up of ART; Model D: Facilities serving as ART referral sites (not providing ANC).

a Facilities (Model A: 2, Model B: 2) excluded as the organization had recently changed from referring women to the ART clinic, to initiating them on ART at the ANC clinic, following MOH recommendations;

b started ART during antenatal period; before or after 27 weeks gestation combined;

c Medical Doctors, Clinical officers, Nurses, Midwives, Medical Assistants.

There were no significant differences across the models in median number of women registered for ANC, median number of known HIV-*positive* or newly HIV test-positive pregnant women, and the proportion of known HIV-*positive* women already on ART per health facility per quarter.

A significant difference was found in the proportion of women who were tested for HIV during ANC, with the lowest proportion in facilities using Model B and the highest in facilities using Model A.

The median number of women per health facility in the most recent six month cohort, i.e. women who started ART (Option B+) between July and December 2012, was 51 (IQR 31–82) with no significant difference between the models. In this cohort, the proportion of women who transferred to another health facility was not significantly different between the models. However, significant differences in retention on ART and defaulting were observed across the models; the highest proportion of defaulting occurred in facilities using Model B and the lowest in Model D facilities.

Observations were similar in the earlier cohort registered between January and June 2012. Across all health facilities, at six months 6% (95% CI 5–8) had transferred out, 15% (95% CI 12–17) had defaulted from care and 84% (95% CI 81–88) were retained on ART. At 12 months, 7% (95% CI 6–9) had transferred out, 17% (95% CI 15–20) had defaulted and 81% (79–84) were retained on ART. At 6 and 12 months, significant differences in retention on ART and defaulting were observed between the models, the highest proportion of defaulting occurred in facilities using Model B and the lowest in Model D facilities.

Client to staff ratios were significantly different across the models, the number of women per clinical staff and HTC counsellors were highest in Model B facilities and lowest in Model C and D facilities.

### Characteristics of health facilities and associations with uptake of HTC at ANC

Table 3 describes the results of univariable and multivariable binary logistic regression analysis, in which we explored associations between characteristics of health facilities and uptake of HTC among pregnant women newly registered for ANC between July 2012 and June 2013.

**Table 3:** Characteristics of health facilities and association with >85% of women with HIV status ascertained during ANC at facility level

			% HIV Status ascertained at ANC				
							
		N	med (IQR)	Crude OR (95% CI)	*p*	Multivariable OR (95% CI)^a^	*p*
District	District 1	17	85 (72–95)	–	–		
	District 2	37	76 (46–83)	**0.2 (0.05–0.7)**	**0.01**		
	District 3	14	79 (73–84)	0.3 (0.06–1.5)	0.1		
	District 4	21	82 (74–90)	1.0 (0.3–3.7)	0.9		
	District 5	11	75 (60–87)	0.6 (0.1–3.0)	0.6		
	District 6	32	83 (71–93)	0.7 (0.2–2.5)	0.7		
Facility type	District hospitals	3	90 (83–92)	–	–		
	Community hospitals	8	82 (67–91)	0.3 (0.02–4.9)	0.4		
	Health centres	117	78 (65–87)	0.2 (0.02–2.5)	0.2		
	Private clinics	4	88 (68–93)	0.5 (0.02–11)	0.6		
Number of women registered in ANC per quarter	<*147*	33	84 (77–91)	–	–		
	*IQR 147–400*	66	78 (62–87)	0.5 (0.2–1.2)	0.1		
	>*400*	33	75 (60–85)	0.5 (0.2–1.2)	0.1		
Number of women registered for ANC per clinical staff^b^	<12	33	84 (77–92)	5.0 (1.6–16)	0.007		
	IQR 12–37	65	80 (66–89)	2.8 (1.0–8.4)	0.06		
	>37	34	72 (55–82)	–	–		
Number of women registered for ANC per HTC counsellor	<13	33	86 (76–94)	34 (4–200)	0.001	44 (4–500)	0.003
	IQR 13–38	66	81 (70–89)	17 (2–131)	0.007	15 (2–132)	0.01
	>38	34	71 (44–79)	–	–	–	–
Number out of 4 quarterly observations “out of stock” of HIV test kits	0	30	82 (78–90)	5.2 (1.0–27)	0.05	3.1 (0.5–20)	0.2
	1	41	84 (74–91)	7.7 (1.6–38)	0.01	7.2 (1.3–50)	0.03
	2	40	77 (63–86)	3.4 (0.7–17)	0.1	2.2 (0.4–14)	0.4
	3 or 4	20	74 (62–82)	–	–	–	–
Model of Option B+ service provision^c^	Model A	73	84 (75–92)	4.6 (1.7–12.4)	0.002	3.4 (1.01–12)	0.05
	Model B	36	74 (55–82)	–	–	–	–
	Model C	18	76 (60–80)	0.6 (0.1–3.2)	0.54	0.3 (0.05–2.8)	0.3

a Controlled for all other variables shown in this table. In the multivariable analysis, variables with significant associations are shown only;

b Medical Doctors, Clinical officers, Nurses, Midwives, Medical Assistants.

c Model A: Facilities where women are initiated and followed on ART at ANC until delivery; Model B: Facilities where women receive only first dose of ART at ANC, and are referred to the ART clinic for follow-up; Model C: Facilities where women are referred from ANC to the ART clinic for initiation and follow-up of ART.

In multivariable analysis, health facility factors independently associated with high uptake of HTC were the number of women registered per HTC counsellor, the number of observations “out of stock” of HIV test kits, and the “model of care” applied.

In comparison to facilities with more than 38 women per HTC counsellor per month (above IQR); facilities with 13–38 (IQR) or less than 13 (below IQR) women per HTC counsellor per month were 15 and 44 times more likely to have high uptake of HTC in ANC [aOR 15(2–132) and aOR 44(4–500)].

Facilities with at most one quarterly observation “out of stock” of HIV test kits were seven times more likely to have high uptake of HTC in ANC than facilities with three or four observations “out of stock” [aOR 7.2 (1.3–50)].

In comparison with Model B, facilities using Model A were three times more likely to have higher uptake of HTC in ANC [aOR 3.4 (1.01–12)].

### Characteristics of health facilities and associations with retention on ART (option B+)

Table 4 describes the results of multinomial univariable and multivariable logistic regression analyses, in which we explored associations between characteristics of health facilities and six-month retention rates for the cohorts of women registered between July and December 2012.

**Table 4:** Characteristics of health facilities and association with retention on ART (Option B + ) at facility level

				Crude OR (95% CI)				Multivariable OR (95% CI)^a^			
						
				reference category 72%–92%				reference category 72%–92%			
						
		N	six-month retention med (IQR)	<72%	*p*	>92%	*p*	<72%	*p*	>92%	*p*
District	District 1	17	71 (67–78)	–	–	–	–	–	–	–	
	District 2	40	79 (64–90)	0.6 (0.2–2.2)	0.5	3.6 (0.4–34)	0.3	1.1 (0.2–5.0)	0.9	5.0 (0.4–67)	0.2
	District 3	14	79 (73–95)	0.2 (0.03–1.1)	0.06	4.3 (0.4–47)	0.2	0.3 (0.04–2.4)	0.3	5.7 (0.3–87)	0.2
	District 4	21	86 (78–90)	0.07 (0.1–0.4)	0.004	0.7 (0.05–9)	0.7	0.06 (0.01–0.41)	0.004	0.6 (0.04–9)	0.7
	District 5	12	90 (80–94)	0.1 (0.01–0.9)	0.04	3.5 (0.3–40)	0.3	0.2 (0.01–2.1)	0.2	5.7 (0.3–99)	0.2
	District 6	31	91 (85–98)	0.1 (0.02–0.6)	0.01	5.2 (0.5–45)	0.2	0.2 (0.03–0.9)	0.04	8.5 (0.7–99)	0.1
Facility type	District hospitals	4	70 (60–88)	–	–	–	–				
	Community hospitals	8	80 (65–86)	0.4 (0.02–6)	0.5	0.3 (0.01–8)	0.4				
	Health centres	115	84 (72–92)	0.3 (0.02–3)	0.3	0.4 (0.03–7)	0.6				
	Private clinics	8	96 (90–99)	–		1.7 (0.1–37)	0.7				
ART/PMTCT services *available and offered*	Certain weekdays	79	86 (74–92)	0.6 (0.3–1.5)	0.3	1.1 (0.5–2.7)	0.8				
	Daily (all weekdays)	51	80 (65–90)	–	–	–	–				
Number of women in	*<31*	33	89 (80–99)	1.3 (0.4–4.8)	0.6	4.1 (1.2–14)	0.03	1.5 (0.3–6.2)	0.6	5.1 (1.2–22)	0.03
six-month cohort	*IQR 31–82*	68	83 (70–91)	2.1 (0.8–5.9)	0.1	2.3 (0.7–7.1)	0.2	2.7 (0.7–12)	0.2	2.2 (0.6–7.6)	0.2
(Jul–Dec 2012)	*>82*	34	81 (75–91)	–	–	–	–	–	–	–	–
Number of women in six month cohort per clinical staff^b^	<7	35	82 (71–99)	0.9 (0.3–3.0)	0.9	1.2 (0.4–3.9)	0.8				
	IQR 7–22	66	88 (73–93)	0.8 (0.3–2.0)	0.6	1.1 (0.4–3.0)	0.9				
	>22	35	81 (75–91)	–	–	–	–				
Adherence	Day of ART initiation	93	85 (72–92)	–	–	–	–				
Counselling	Next visit	14	87 (66–95)	2.4 (0.6–10)	0.2	2.4 (0.6–9)	0.2				
	Both same day and next visit	24	86 (76–91)	0.6 (0.2–1.9)	0.4	0.3 (0.1–1.3)	0.1				
Availability of ART/MIP clinic for follow-up	MIP clinic	29	78 (67–89)	–	–	–	–				
	ART clinic	106	86 (75–93)	0.5 (0.2–1.2)	0.1	1.8 (0.5–6)	0.4				
Model of Option B +	Model A	73	83 (70–91)	1.0 (0.4–2.5)	0.9	1.7 (0.5–5.3)	0.4	0.9 (0.3–3.4)	0.9	3.0 (0.7–12)	0.1
service provision^c^	Model B	36	80 (70–87)	–	–	–	–	–	–	–	–
	Model C	18	91 (82–96)	0.5 (0.09–3)	0.5	4.6 (1.2–19)	0.04	0.4 (0.05–3.4)	0.4	5.4 (1.2–28)	0.04
	Model D	9	95 (90–99)	0.9 (0.07–11)	0.9	8.0 (1.1–57)	0.04	0.9 (0.06–16)	0.9	9.1 (0.9–84)	0.06

a Controlled for all other variables shown in this table. In the multivariable analysis, variables with significant associations are shown only.

b Medical Doctors, Clinical officers, Nurses, Midwives, Medical Assistants.

c Model A: Facilities where women are initiated and followed on ART at ANC until delivery; Model B: Facilities where women receive only first dose of ART at ANC, and are referred to the ART clinic for follow-up; Model C: Facilities where women are referred from ANC to the ART clinic for initiation and follow-up of ART; Model D: Facilities serving as ART referral sites (not providing ANC).

In multivariable analysis, health facility factors independently associated with low or high six-month retention were district location, number of women in the cohort, and the “model of care” applied.

Health facilities in districts 4 and 6 were less likely to have lower retention rates than health facilities in other districts, independent of other health facility characteristics.

Facilities with less than 31 women (below IQR) in the six-month cohort were five times more likely to have high retention rates than facilities with more than 82 women (above IQR) in the cohort [aOR 5.1 (1.2–22)].

In comparison with Model B, facilities using Model C were five times more likely to have higher retention rates [aOR 5.4 (1.2–28)].

## Discussion

In Malawi, the Option B+ strategy has been scaled up with major increases in HIV-positive women starting ART during pregnancy [4,9]. Our study shows that a large variation exists in the way health facilities in the South East Zone of Malawi have integrated PMTCT Option B+ care into their routine service delivery. We identified four models of PMTCT care provision and found that these “models of care” were associated with uptake of HTC at ANC clinics and retention in care on ART. We observed that worse program indicators were found in Model B facilities in which women receive the first dose of ART at the ANC clinic and are then transferred to the ART clinic for ART follow-up. In these facilities, 32% of women did not receive an HIV test at ANC and 20% of women who started ART under Option B+ had defaulted six months after initiation.

Following current Ministry of Health recommendations, several health facilities have recently changed their practice from referring women to the ART clinic to initiating them on ART at the ANC clinic. However, our findings suggest that such a change may only be beneficial if identified HIV-positive pregnant women are initiated and followed on ART at the ANC clinic until delivery. We found that health facilities that opted for Model B also had highest client–staff ratios, which may have driven the choice of approach. In such situations, referral to an ART clinic for initiation and follow-up of ART may seem more efficient.

Our study stresses the need for further patient-level research to ascertain which models of care are most successful in having women accept initiation and lifelong continuation of ART. It has been reported that pregnant women who initiated ART on the day of diagnosis were almost twice as likely not to return for follow-up after the initial visit [5]. In such circumstances, women may be insufficiently able to comprehend and accept the full consequences of a positive HIV test and the need to start lifelong ART all at once.

Despite the apparent availability of HTC in ANC settings, many pregnant women still do not get tested, and thereby miss a potential opportunity to prevent vertical transmission to their infants and care for their own health. A recent study that we conducted in Malawi reported that some mothers chose not to get tested for fear of stigmatization and that the main system-related barriers to getting tested during pregnancy were lack of available resources and women not being offered a test [13]. Facilities where HIV-positive women were initiated and followed on ART at the ANC clinic (Model A) had significantly lower proportions of untested women than facilities where women were referred to the ART clinic or were initiated on ART in ANC and then referred to the ART clinic. It is possible that when ANC personnel are fully involved in providing ART until delivery, there may be a greater recognition of the importance of identifying women in need of ART. We found that in some facilities ART adherence counselling was first conducted at the first visit after ART initiation. It is likely that in those facilities some women who started ART had not made a fully informed choice to start and adhere to treatment and were consequently more likely to stop after their first dose of ART received in the ANC clinic. In facilities where pregnant women are diagnosed with HIV at ANC and then referred to the ART clinic for initiation, they probably have more time for reflection, consider disclosure of their status to their partner, and may therefore be better prepared to start ART [14–16]. Optimal timing and quality of counselling and whether time between HIV diagnosis and ART initiation affect retention on treatment are priorities for further operational research.

We found no differences in treatment outcomes between the earlier and most recent six-month cohorts, suggesting that the effects of health facility variables are stable over time and not the result of start-up problems in a new situation. There was no notable loss in retention between six and 12 months follow-up. This is consistent with findings from a recent national study indicating that most losses among newly started pregnant women were observed in the first three months on ART and in particular before the first follow-up visit [5].

The difference that we found in six-month retentions between districts could be explained by variation in district health management capacity, a different level of non-governmental organization support, or ethnic and cultural differences that may influence ART uptake and compliance.

Our finding that low retention rates are associated with larger patient volumes at health facilities confirms earlier observations [5], and may be related to less favourable health-system factors in larger ART programs, such as patient burden per staff member and longer waiting times.

Several limitations of our study need to be considered. We studied a large sample of Malawi health centres, but our results may not be representative for health facilities outside the South East Zone. We were not able to analyse variables at individual patient level, which may have differed between health facilities and across models of PMTCT care. Although we used multivariable analysis to study the association of models of PMTCT care with retention on ART, health facilities are likely to have made decisions about the organization of PMTCT care on the basis of factors related to their specific circumstances leaving a potential for residual confounding. We did not study attrition that may have occurred during linkage of care before the start of ART, which is particularly applicable to Models C and D, and may have resulted in selection bias.

## Conclusions

A large variety exists in the way health facilities have integrated PMTCT Option B+ care into the routine service delivery. This study shows that the “model of care” that health facilities chose to achieve this integration is associated with uptake of HTC in ANC and retention in care on ART. Further patient-level research is needed to ascertain whether time between HIV diagnosis and ART initiation affect retention in care and to determine which specific service delivery models are most successful in promoting initiation of ART and achieving adherence to lifelong treatment.
